# An Evaluation Service for Digital Public Health Interventions: User-Centered Design Approach

**DOI:** 10.2196/28356

**Published:** 2021-09-08

**Authors:** Kassandra Karpathakis, Gene Libow, Henry W W Potts, Simon Dixon, Felix Greaves, Elizabeth Murray

**Affiliations:** 1 NHS Artificial Intelligence Laboratory NHSX London United Kingdom; 2 Independent Service Design Consultant London United Kingdom; 3 Institute of Health Informatics University College London London United Kingdom; 4 NHSX London United Kingdom; 5 Department of Primary Care and Public Health Imperial College London London United Kingdom; 6 Science, Evidence and Analysis National Institute of Health and Care Excellence London United Kingdom; 7 Research Department of Primary Care and Population Health University College London London United Kingdom

**Keywords:** digital health, internet-based interventions, mHealth, evaluation studies, public health, human-centered design, service design, mobile phone

## Abstract

**Background:**

Digital health interventions (DHIs) have the potential to improve public health by combining effective interventions and population reach. However, what biomedical researchers and digital developers consider an effective intervention differs, thereby creating an ongoing challenge to integrating their respective approaches when evaluating DHIs.

**Objective:**

This study aims to report on the Public Health England (PHE) initiative set out to operationalize an evaluation framework that combines biomedical and digital approaches and demonstrates the impact, cost-effectiveness, and benefit of DHIs on public health.

**Methods:**

We comprised a multidisciplinary project team including service designers, academics, and public health professionals and used user-centered design methods, such as qualitative research, engagement with end users and stakeholders, and iterative learning. The iterative approach enabled the team to sequentially define the problem, understand user needs, identify opportunity areas, develop concepts, test prototypes, and plan service implementation. Stakeholders, senior leaders from PHE, and a working group critiqued the outputs.

**Results:**

We identified 26 themes and 82 user needs from semistructured interviews (N=15), expressed as 46 Jobs To Be Done, which were then validated across the journey of evaluation design for a DHI. We identified seven essential concepts for evaluating DHIs: evaluation thinking, evaluation canvas, contract assistant, testing toolkit, development history, data hub, and publish health outcomes. Of these, three concepts were prioritized for further testing and development, and subsequently refined into the proposed PHE Evaluation Service for public health DHIs. Testing with PHE’s Couch-to-5K app digital team confirmed the viability, desirability, and feasibility of both the evaluation approach and the Evaluation Service.

**Conclusions:**

An iterative, user-centered design approach enabled PHE to combine the strengths of academic and biomedical disciplines with the expertise of nonacademic and digital developers for evaluating DHIs. Design-led methodologies can add value to public health settings. The subsequent service, now known as *Evaluating Digital Health Products*, is currently in use by health bodies in the United Kingdom and is available to others for tackling the problem of evaluating DHIs pragmatically and responsively.

## Introduction

For public health interventions to significantly impact healthy life expectancy in a population, reach has to be combined with efficacy [1]. Reach is the proportion of the target population affected by an intervention, and efficacy is the effect of an intervention on the individuals it reaches. There is considerable interest in the potential of digital health interventions (DHIs) to improve public health. DHIs are delivered on digital platforms, such as the web or smartphone apps intended to deliver health care or health promotion [2]. Such DHIs are expected to combine the reach of large-scale population initiatives, such as media advertising, with the efficacy of individual treatments. Furthermore, DHIs are intended to increase both capacity and access to public health initiatives by providing services in areas where face-to-face options are unavailable or unable to meet demand.

Although there is evidence for the efficacy of many DHIs in public health [3-10], few studies indicate that their public health potential is being realized in practice. What constitutes success is viewed differently by biomedical researchers and digital product developers. Biomedical research on digital health is heavily influenced by the pharmaceutical model with a focus on trials and effectiveness, whereas digital developers often focus on usability and acceptability [11]. However, both perspectives are required [11]. A digital product cannot be successful at scale without satisfactory usability. However, user ratings can be insufficient, sometimes bearing either no relation or even an inverse relation to the effectiveness of DHIs [12-14].

As a public health body with a finite budget and responsibility for improving population health, Public Health England (PHE) was at the forefront of considering how best to use DHIs to improve health outcomes and evaluate their value (measured as an improvement to public health). Multiple biomedical and digital approaches to the evaluation of DHIs exist and are described and critiqued elsewhere [2,11,15-18]. This paper reports on a project by PHE to develop and operationalize an evaluation framework that combines these approaches with the goal of demonstrating the impact, cost-effectiveness, and benefit of DHIs on public health.

## Methods

### Design

User-centered design (UCD), applied by a multidisciplinary team, was used to synthesize the strengths of digital evaluation approaches with those of the biomedical approach. The project objectives were as follows:

1. Identify core audiences and stakeholders for evaluation of DHIs (user groups)
2. Identify the needs of users for evaluating DHIs, including barriers and facilitators
3. Identify the key performance indicators (KPIs) and outcomes that different audiences and stakeholders consider important in evaluating DHIs
4. Identify evaluation methods (ways of conducting an evaluation study), tools (resources that can aid in carrying out an evaluation), and metrics (standard quantitative measures that can be used in an evaluation) applicable to DHIs
5. Prototype and test an evaluation approach for DHIs used in a public health context

### User-Centered Design

A modified UCD approach, known as service design, was adopted. The UCD approach bases the design, testing, and development of a product or service on the needs of the users affected by it [13]. This approach, which began as a focus on the end user’s interaction with products, has evolved into designing both products and services. It considers the needs of a broad range of users, including organizational users providing the service, users responsible for the finances and direction of the service, and other users in the service ecosystem. This holistic evolution is reflected in service design [19,20], a choreography of the processes, technologies, and interactions needed to set up and drive a service through a UCD perspective [21].

Service design helps reduce risk by investing upfront in validating a service proposition with end users before physically building the product or service (desirability and usability), clarifying what is required of the organization to deliver the service (feasibility), clarifying the potential business impact of the service (viability), having a strong focus on outcomes, and embedding the host organization’s team that will eventually run the service in research and design activities [22]. By designing to meet the needs of users, service design methods are intended to ensure that the resultant service is more likely to be desirable, usable, feasible, and viable. Throughout this study, the term UCD refers to the service design variation.

The project team followed the English Government Digital Service’s agile approach, comprising discovery, alpha, beta, and live phases [23]. This approach has subsequently been adopted by governments worldwide. This paper reports the discovery and alpha phases.

### Setting and Project Team

At the time of the project, PHE was the national public health agency in England responsible for protecting and improving the nation’s health and well-being and reducing health inequalities. The discovery phase was delivered from May to June 2018 and the alpha phase was delivered from August 2018 to March 2019. The beta phase commenced in July 2019 and was completed in 2021.

The project team was established using a competitive tender [19], where applicants read a seminal paper [2] about evaluating DHIs and presented an example of how they would integrate evaluation into the design and development of a DHI. The successful applicant, Livework Studio Ltd, a service design agency, worked alongside the internal PHE team to form the overall project team (the project team).

In response to this challenge, Livework created a visual model showing how different evaluation approaches and metrics could be integrated into the design and experience of a DHI. The model or design object (Figure S1 in Multimedia Appendix 1) tangibly represents the problem of evaluating DHIs for various stakeholders. The model was iterated as the project progressed.

PHE formed a working group with representatives across the English health system, including the Department of Health and Social Care, National Institute of Health and Care Excellence (NICE), University College London (UCL), and the World Health Organization. The working group provided knowledge on the wider digital health landscape and sense-checked research and deliverables.

The project team conducted 14 *show and tells* in the discovery and alpha phases, wherein the project’s progress was shared with a wider group of stakeholders (*Show and tells* are opportunities for team members to showcase their work to their team and wider stakeholders; it is a project management and engagement technique commonly used by digital professionals). This included sharing project plans, early research findings, and design outputs from different research sessions. Stakeholder feedback helped validate or raise concerns with the findings and linked the project team to initiatives in the wider digital health ecosystem.

### Ethics

This project complied with the code of conduct of the Market Research Society as required by PHE’s Research Ethics and Governance Group, and informed consent was obtained from the interview participants. The project’s methodology and results were presented at two government-mandated service assessments [23] at the end of the discovery and alpha phases to ensure adherence to the government Service Standard [24] for digital projects.

### Recruitment

Three categories of professionals involved in the design, development, and commissioning of public health DHIs were identified: academics, digital product developers (hereafter referred to as *digital developers*), and public health professionals. Research participants (N=15) for the interviews were selected to reflect these user groups. Additional participants were recruited in the following stages.

Academics were recruited for their expertise in developing and evaluating DHIs, as reflected in their publications and national reputation. Digital professionals with a track record of successful development and wide-scale deployment of DHIs were selected, including those working for PHE, National Health Service England and Improvement (NHSE/I), and the private sector. Public health professionals were selected for their experience of commissioning or delivering public health services via digital means and their strategic overview of the challenges involved. Within digital and public health professionals, those with and without experience of evaluating DHIs were sampled.

## Results

### Iterative Data Collection Methods Presented With Results

**Figure 1:**
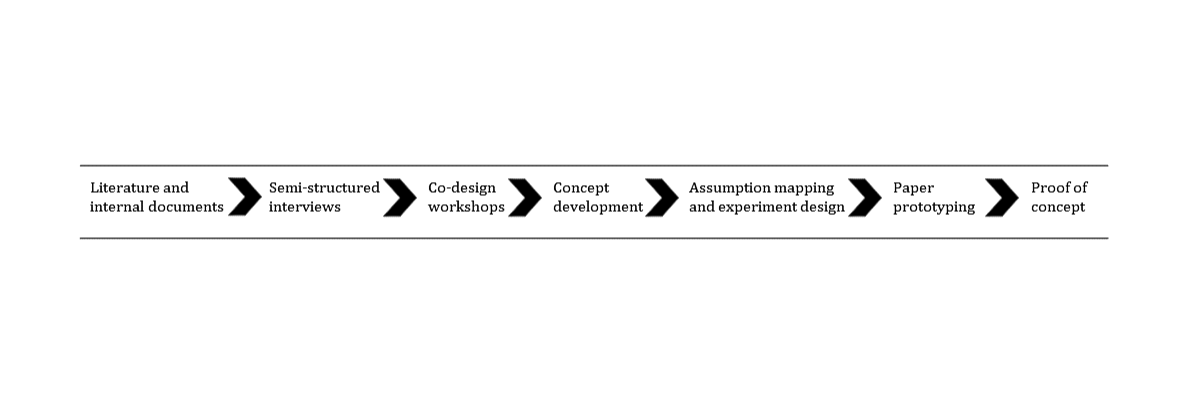
The seven data collection methods with the output of each stage becoming the input for the next stage.

We used seven data collection methods: (1) review of the literature and internal documents, (2) semistructured interviews, (3) co-design workshops, (4) concept development, (5) assumption mapping and experiment design, (6) paper prototyping, and (7) proof of concept. The outputs of each stage were the inputs for the next stage (Figure 1). Through a series of iterative developments, the project team progressively evolved its understanding of the problem, user needs, and potential solutions. The seven data collection and analysis methods, along with the results of each stage are presented in the following sections.

### Review of the Literature and Internal Documents

#### Methodology

Internal PHE documents and sample publications on the evaluation of DHIs provided by PHE staff, including the paper by Murray et al [2] used in the supplier challenge, were reviewed. The aims of the review were to (1) develop an understanding of the problem space, (2) identify themes pertaining to evaluating DHIs—including barriers and facilitators—for exploration in semistructured interviews (project objective 2), and (3) identify evaluation methods, tools, and metrics applicable to DHIs (project objective 4). The evaluation methods identified were categorized into academic, health, economic, and digital methods. For each method, a simple description, scenarios of use, pros and cons, cost, time, and existing tools and guidance were captured.

The output of stage 1 formed the basis of a discussion guide (Multimedia Appendix 2) for the semistructured interviews (stage 2). Additional publications were collected and synthesized throughout the study.

#### Corresponding Results

The barriers identified were organizational and disciplinary silos associated with public health commissioning, design and development of digital services, and academic evaluation; unclear expectations and roles; different disciplinary expectations on what tools and methods to use for evaluation and how to measure success or failure; limited time, capacity, and funding; lack of evaluation experience; and rapidly evolving DHIs (inherent tension with resource- and time-intensive evaluations).

The facilitators identified were clear organizational goals (outcomes) and decision-making processes, prioritizing evaluation from the outset, ring-fenced funding, team capacity and resources, guidance and case studies, communities of practice, interdisciplinary collaboration, data standards, and constructive feedback.

The resultant discussion guide (Multimedia Appendix 2) was structured around the process of conducting an evaluation of a DHI, as identified from the review of the literature and internal PHE documents. Discussion guide sections included unawareness and initiation of an evaluation; setup, information, guidance, and tools; data, decisions, and outputs; and wrap up, outputs, and outcomes. Questions within each section were created to further explore themes pertaining to evaluating DHIs, including decision-making; intervention mechanisms of action; organizational obligations (to evaluate); metrics; success criteria; and feedback loops. For the different user groups, additional questions exploring the barriers to and facilitators of evaluating DHIs were added. In total, 46 evaluation methods were identified and categorized (Textbox 1; additional information captured is available upon request).

**Evaluation methods identified from the review of literature and internal documents.**

**Academic**

- Cluster randomized controlled trial (RCT)
- Consultative or deliberative methods
- Documentary analysis
- Ethnography
- Factorial RCT
- Feasibility RCT
- Focus group
- Individual and group interviews
- Interrupted time series
- Natural experiment
- Nonrandomized controlled before and after
- Process evaluation
- Propensity score-matched control study
- RCT
- Sequential multiple assignment randomized trial
- Stepped-wedge RCT
- Survey of digital health intervention users
- Surveys of target population for digital health intervention
- Uncontrolled before and after
- Qualitative comparative analysis
- Qualitative evaluation
- Quantitative observation studies

**Digital**

- A/B testing
- Advertising data
- App Store data
- Cohort analysis
- Digital uptake
- Email tracking
- Funnel analysis
- Guerrilla user research
- In app analytics
- Likes or shares
- Net promoter score
- Microrandomized trials
- Notifications
- Web-based survey or polling
- Search traffic
- Self-reporting
- Simulation modeling
- Social media research
- Usability testing (laboratory based)
- Wearable device data
- Web analytics

**Health Economic**

- Cost-benefit analysis
- Cost consequence
- Cost-effectiveness analysis
- Cost utility

### Semistructured Interviews

#### Methodology

Semistructured interviews were conducted to identify user needs (project objectives 1 and 2). Interviews explored participants’ roles in relation to commissioning, designing, or developing DHIs; their understanding of evaluation; and their previous evaluation experience. Subsequent questions focused on the process of evaluation and its evolution as a DHI are developed, along with the key themes, barriers, and facilitators identified in stage 1.

Interviews were conducted by service designers with expertise in design research, lasted 60-90 minutes, and were audiorecorded. Detailed field notes were also provided. Both field notes and transcribed interviews formed a part of the data set.

An interview capture template was used to highlight and thematically organize the interview data. After each interview, the researcher reviewed their field notes and transcripts, highlighting the points of interest. These points were coded using a common set of themes based on the stages of the evaluation process (before, beginning, during, and after) and known areas of interest, such as collaboration, clinical risk, support and guidance, technology, and data, as identified in stage 1. If an observation did not obviously fit within an existing theme, a new theme was created to code it, or it was highlighted for further review by the wider project team.

Key user statements and interview observations generated the first iteration of user needs [18], including needs being met by current means of evaluating DHIs and needs that remained unmet or were causing difficulty and frustration in evaluation efforts. Identified user needs were articulated using an accepted UCD template called Jobs To Be Done (JTBD), which identifies the type of user, their need, when the need arises, and the outcome they are trying to achieve [25]. This structured articulation clearly communicates the user’s needs to stakeholders and the project team.

After the initial round of analysis, the project team collectively reviewed all interviews; shared points of interest, observations, and user needs; and clustered them according to the common themes. User needs were mapped against a common user journey—a way of showing a user’s journey through a service across time [26]. This produced an overview of where and when user needs occurred throughout the process of designing a DHI evaluation.

#### Corresponding Results

A total of 15 semistructured interviews were completed, five per target user group (Table 1). Some participants were invited to take part in multiple data collection stages, with 6 interviewees participating in the co-design workshops (stages 3 and 4), 4 co-design workshop attendees participating in paper prototyping (stage 7), and 1 interviewee participating in the proof of concept (stage 8).

In total, 26 themes about evaluating DHIs were identified and validated (Textbox S1, Multimedia Appendix 1). These themes were organized by a user group (academic, digital, and public health) and an evaluation stage (overview; unawareness and initiating an evaluation; setup, information, guidance, and tools; data, decisions, and outputs; outcomes, feedback, and decisions; and incidents and changes).

A total of 82 JTBD were generated (18/82, 21% academic; 37/82, 45% digital; and 27/82, 32% public health), derived from the outputs of stages 1 and 2. Table 2 shows an example of the semistructured interview analysis and the resulting JTBD.

The initial set of JTBDs was refined and augmented through feedback from stakeholders at the show and tells. The project team then reviewed the outputs and distilled the superset of research findings and user needs into a smaller subset of user needs or JTBD: (1) representing the essential stages of the journey, (2) most essential stages (ie, if the need was not met then subsequent needs could not be met), and (3) those stages most strongly indicated by research stages 1 and 2 (semistructured interviews and publications). The refined 46 JTBDs (13/46, 28% academic; 20/46, 43% digital; and 13/46, 28% public health) and the outputs of stage 1 formed the input for the first co-design workshop.

**Table 1:** Overview of participant characteristics at data collection stages 2, 3, 4, 7, and 8.

Participants			Semistructured interviews (stage 2; n=15)		Co-design workshop 1 (stage 3; n=10)		Co-design workshop 2 (stage 4; n=10)		Paper prototypes (stage 7; n=11)		Proof of concept (stage 8; n=6)
**User type, n (%)**											
	Academic	5 (33)		1 (10)		3 (30)		2 (18)		N/A^a^	
	Digital	5 (33)		5 (50)		4 (40)		6 (55)		6 (100)	
	Public health	5 (33)		4 (40)		3 (30)		3 (27)		N/A	
Organizations			Be Mindful Fresh Egg Lancashire County Council Mindtech NHSE/I^b^ PHE^c^ SH:24 The University of Edinburgh UCL^d^ WHO^e^		Mindwave PHE SH:24 UCL		DHSC^f^ King’s College London NICE^g^ PHE Unboxed WHO		DHSC PHE		PHE

^a^N/A: not applicable.

^b^NHS E/I: National Health Service England and Improvement.

^c^PHE: Public Health England.

^d^UCL: University College London.

^e^WHO: World Health Organization.

^f^DHSC: Department of Health and Social Care.

^g^NICE: National Institute of Health and Care Excellence.

**Table 2:** For each user group, we provide an example aligned theme, illustrative quote, and Jobs To Be Done mapped to the relevant evaluation stage.

User group	Evaluation stage	Theme	Illustrative quote	Jobs To Be Done
Academic	Stage 2: setup, information, guidance, and tools	Evaluation methods	“You have a set of tools and paradigms that are going to be suitable for different types of problems. When you’re investigating different types of problems, for particular types of stakeholders, which particular types of objectives, or even particular types of technology, or particular stages of innovation, you have to be careful about finding the right thing and not trying to squeeze a round peg into a square hole.”	As an evaluator When I design/set up an evaluation I need access to a range of evaluation tools and paradigms So that I can choose the one fit for the problem
Digital	Stage 4: outcomes, feedback, and decisions	Design process	“It’s fundamental to my role in a non-academic non-traditional sense, as far as a non-empirical sense, because I'm the one who manages [metric company name] and given what it takes to insure and also the research we conduct with users to define and validate services prior to committing resources developing them. But also to maximize them later, so we use informal or design led evaluation means to validate, to research, to prove assumptions prior to designing things.”	As a digital professional When deciding what to design and how to design it I need to validate service propositions by proving assumptions So that I can be confident in committing resources developing them
Public health	Stage 0: overview	Funding and costs	“First, we’re using it to channel shift. So, if we can get people to use digital interventions rather than face-to-face interventions which are much more expensive, then we’re developing a range of products and services around that. On sexual health, we have a new program on contraceptive access for women to help them to make choices online and to get their contraceptive online rather than going in through GP^a^ services.”	As a Director of Public Health and Well-being When I am planning service changes I need to know if people will use a digital approach So that we can save money by shifting service from more expensive face-to face-services

^a^GP: general practitioner.

### Co-design Workshop 1

#### Overview

Two co-design workshops were held to (1) validate the findings from semistructured interviews (project objectives 1 and 2), (2) help create a user journey that reflected the user requirements for all three user groups (project objective 2), (3) identify evaluation methods, tools, and metrics applicable to DHIs (project objectives 2, 3, and 4), and (4) consider the structure of DHIs (ie, intervention component and delivery component; project objectives 4 and 5).

#### Methodology

Findings from stages 1 and 2 were articulated as JTBDs or user needs for each of the three user groups. These were mapped onto a user journey reflecting all stages for meeting the goal of evaluating a DHI (Figure S2; Multimedia Appendix 1). This mapping was used to reflect the timings and interdependencies of each step and the user needs preceding the evaluation of a DHI.

Workshop participants were divided into groups of three: one person from each profession (digital, public health, and academic). These trios worked through the draft user journey from the perspective of a specified user group, discussing reactions, validating the user needs that they recognized, editing where necessary, and adding missing user needs. The exercise was repeated twice until each trio reviewed the draft journey from the perspective of all three user groups. Reviewing all three sets of user needs promoted the understanding of other perspectives among the workshop participants and the project team.

Participants then reviewed and edited a catalog of 46 evaluation methods and tools collated by the project team in stages 1 and 2. Participants considered different ways of categorizing them according to the timing in the journey of planning and conducting an evaluation.

#### Corresponding Results

Workshop participants validated, refined, or rearticulated the 46 JTBDs (13/46, 28% academic; 20/46, 43% digital; and 13/46, 28% public health) across the stages of the evaluation journey. A further 27 JTBDs (8/27, 29% academic; 1/27, 3% digital; and 18/27, 66% public health) were added by workshop participants. The project team had a total of 73 JTBDs (21/73, 28% academic; 21/73, 28% digital; and 31/73, 42% public health) at the end of the first workshop.

The first co-design workshop structure also exposed participants representing the three user groups to the aims, perspectives, and corresponding needs of the other user groups. Participants were observed sharing points of view, articulating their needs that were not self-evident to other user groups, and learning where their respective needs coincided. Furthermore, the different user groups learned about the scope and responsibilities of each other’s roles and where interdependencies between their needs and evaluation activities were.

Workshop participants reviewed, validated, and differentiated the evaluation catalog into evaluation methods, tools, or metrics. Of the initial 46 evaluation methods, tools, and metrics, six were amended, and the participants added three methods, three tools, and 14 metrics.

Participants were observed learning about evaluation methods, tools, and metrics they had not previously used, including their benefits and potential scenarios of use. Participants from different user groups shared with each other how they used the evaluation methods, tools, and metrics in their own role and how they chose the appropriate one, for example, based on the potential health risk of a DHI.

After the first co-design workshop, the project team collectively reviewed the outputs and distilled the 73 JTBDs (21/73, 28% academic; 21/73, 28% digital; and 31/73, 42% public health) into a smaller subset to be used as design stimulus for the second co-design workshop. Following the same process for selecting a subset of user needs as performed in stage 2, the subset of JTBDs was selected by (1) representing the essential stages of the journey, (2) most essential need (ie, if the need was not met then subsequent needs could not be met), (3) those needs most strongly indicated by research stages 1-3, and (4) those needs that were actionable and useful as stimuli for a design workshop.

The project team prioritized 9 JTBDs (3/9, 33% academic; 3/9, 33% digital; and 3/9, 33% public health; Table 3) for input into the second co-design workshop. The resultant catalog of evaluation methods, tools, and metrics from the first co-design workshop formed the basis of an evaluation method catalog (project objective 4) used in stage 8 (proof of concept: prototype of full-service experience).

**Table 3:** The nine Jobs To Be Done prioritized for co-design workshop 2, with three prioritized per user group.

User group	JTBD^a^ 1	JTBD 2	JTBD 3
Public health	As a public health professional When reviewing the outcomes of an evaluation I need the flexibility within the project and team to make decisions that change the direction of the project So that learnings can be used to change the service for the better	As a public health professional When evaluating a service I need to know if the service is having an impact on key metrics So that I can make an investment case for rolling the service out at scale	As a commissioner When writing a brief I need to set expectations around metrics, methods, and implementation as well as provider skills and capabilities So that providers can build these into their solution design and implementation plan
Academic	As an evaluator When evaluating a health product or service I need access to clean, accessible and linked data from across the health system So that I can do my evaluation	As an evaluator When designing or setting up an evaluation I need access to a range of evaluation tools and paradigms So that I can choose the one fit for the problem	As an academic evaluator When doing evaluation I need an in-depth understanding of the intervention and the pathway of action So that I can properly evaluate it
Digital	As a digital professional When evaluating a service qualitatively I need to be able to observe users So that I can understand why they are having difficulties	As a digital professional When planning a DHI^b^ I need to know what sort of evaluations might need to take place So that I can be prepared to participate	As a digital professional When deciding what to design and how to design it I need to validate service propositions by testing assumptions So that I can be confident in committing resources to develop them

^a^JTBD: Jobs To Be Done.

^b^DHI: digital health intervention.

### Co-design Workshop 2

#### Methodology

The second co-design workshop was built on the first and aimed at designing concepts for an evaluation framework for DHIs in a public health context (project objective 5). Attendees worked in groups of three, as before. Using a DHI case study, the attendees created a concept for an Evaluation Service by imagining how a user would conduct an evaluation in each case. Stimulus materials, comprising interactive design artifacts and JTBD from stages 1 to 3 and those specifically created for the workshop (Figure S3 in Multimedia Appendix 1), provided some structure for creating the concept. Attendees synthesized these inputs into a service concept that they named, described, and visualized using a concept template (Figure S3 in Multimedia Appendix 1). Each concept was shared with all workshop attendees for feedback.

**Figure 2:**
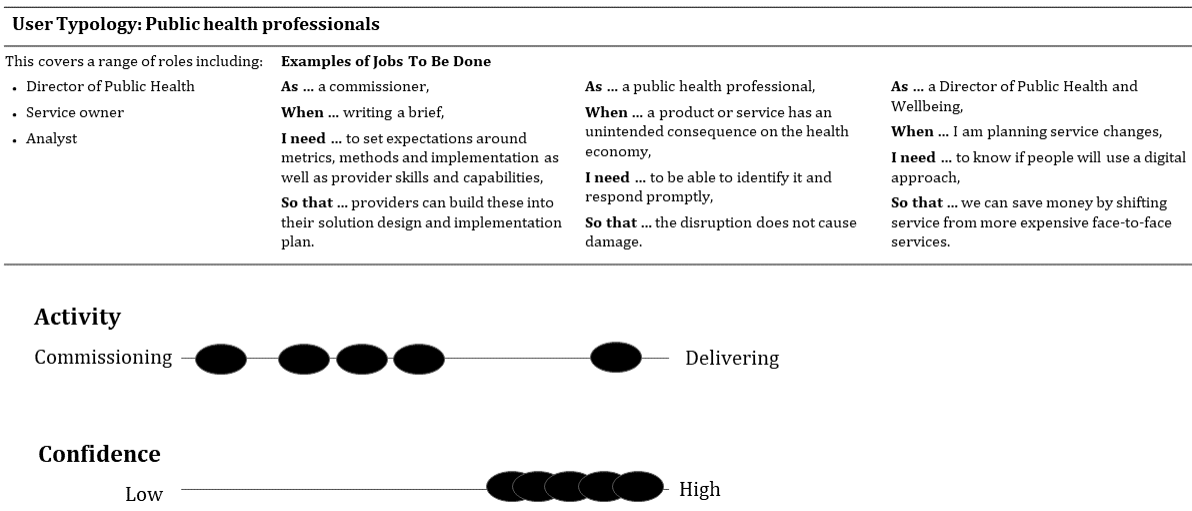
User typology for public health professionals evaluating a digital health intervention (black circles indicate the distribution of participants in stage 2 semistructured interviews).

Workshop outputs included a series of eight conceptual drawings and descriptions. Using the outputs from stages 1 to 4, the project team further articulated the different user groups as user typologies [27] based on key dimensions of difference (Figure 2).

#### Corresponding Results

The workshop produced eight raw concepts considered essential by the participants for the DHI evaluation framework:

1. Integrated evaluation: builds in evaluation thinking, skills, and tools at the beginning of the DHI development process.
2. Parallel playbook: a series of experiments to validate DHI propositions and iterate these to validate the impact.
3. Contract guidance: a person to help write contract terms, thereby ensuring providers build evaluation into the design and agree to cease or change course if the desired outcomes are not indicated.
4. Measurements aggregator: a way to discover and integrate health outcome measures into one place. This would enable digital teams to explore health outcome data and make informed decisions when developing their DHI.
5. Standardized way of collecting or structuring data: a visible, accessible history of the development process for a DHI and how it was evaluated.
6. Conventional medical model: linking user data from a DHI to how it affects end users’ health outcomes.
7. Access shortcuts: an NHS data owner who has an agreement with the local authority and clinical commissioning group for sharing and accessing data to inform DHI development and evaluation.
8. Innovation framework: a PHE open-source innovation framework for understanding the DHI context and sharing data.

Figure 2 shows the user group typologies designed using the dimensions of activity in evaluation (commissioning or delivering) and confidence in evaluation (low or high).

### Concept Development and Prioritization

#### Methodology

**Figure 3:**
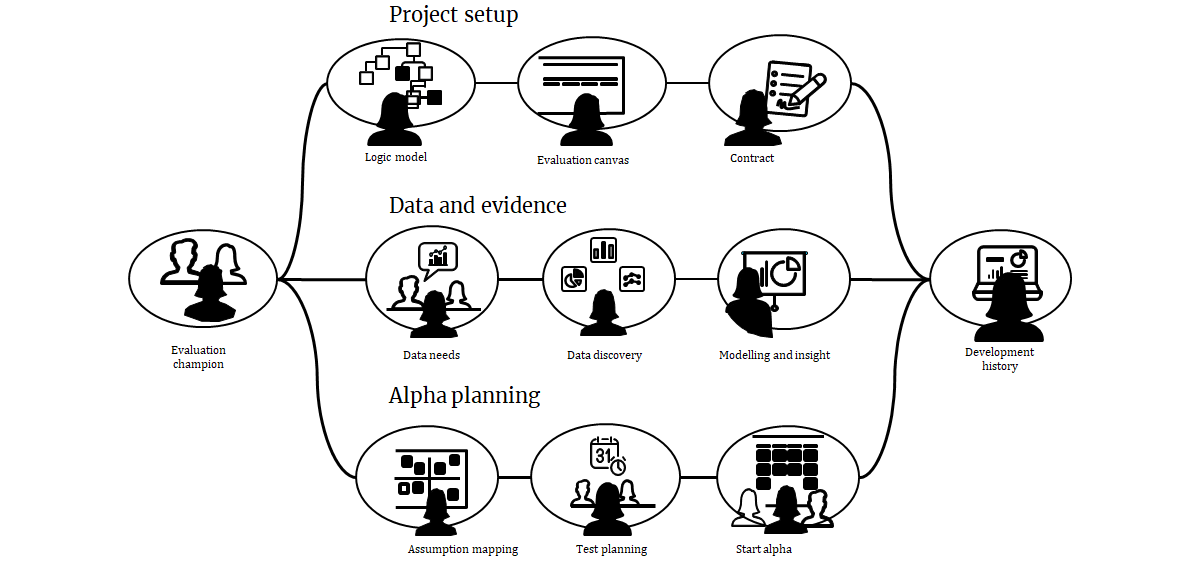
Scenario of use illustrating how the final concepts fit together to facilitate setting up digital health intervention evaluations from conception (project setup) through collection of data and evidence to planning testing of minimum viable products (evaluation planning).

Stages 1 to 4 outputs formed the basis of the concepts the project team further developed, prioritized, and carried forward. We synthesized seven distinct concepts for evaluating DHIs with clear propositions to support prioritization by the project team and working group (Table 4; Multimedia Appendix 3, containing the image of project team member synthesizing stages 1-4 outputs into the seven distinct concepts for evaluating DHIs). How each concept was linked to meet user needs via three key interconnected *scenarios of use* was visualized (Figure 3).

The concepts were scored and prioritized according to the following criteria: meeting user needs, delivering on project objectives, and feasibility for implementation within the PHE. The highest scoring concepts were taken forward into the alpha phase of the project.

**Table 4:** Final seven concepts for evaluating digital health interventions and underlying Jobs To Be Done.

Concept	Concept proposition	Example underlying JTBD^a^	Sample user quote^b^
Evaluation thinking	When doing a digital health project at PHE^c^, evaluation thinking, skills, and tools should be integrated into the project from the start. Evaluation needs to be a central part of the design process and iterative delivery of any PHE service or product.	As a Director of Public Health When commissioning services I need evaluation to be aligned closely with service delivery So that it is formative and not a separate piece of work	“You have to get them at the beginning. And it’s really hard when you don’t get them at the beginning, ‘cause then you’ve got to try and do a retrospective evaluation. You never have the right data. It’s never as good of quality.”
Evaluation canvas	The evaluation canvas is PHE’s validated and accepted portfolio of metrics, tools, and methods for evaluating DHIs^d^. This canvas is the first step to creating a knowledge base on the effectiveness of digital health in meeting health outcomes and will support decisions on policy, practice, and research.	As an evaluator When designing or setting up an evaluation I need access to a range of evaluation tools and paradigms So that I can choose the one fit for the problem	“You have a set of tools and paradigms that are going to be suitable for different types of problems. When you’re investigating different types of problems, for particular types of stakeholders, which particular types of objectives, or even particular types of technology, or particular stages of innovation, you have to be careful about finding the right thing and not trying to squeeze a round peg into a square hole.”
Contract assistant	A way for PHE teams to create strategic relationships with suppliers, supported by forward-thinking contracts. A core requirement when working for PHE will be embedding evaluation into the design and development of the DHI and allowing for flexibility in contracted deliverables as the DHI progresses.	As a public health professional When setting up evaluations I need to get the data-sharing agreements in place as simply as possible So that I can collect, collate, and evaluate the data	“It’s setting a level of expectation...as part of their bids, they need to articulate how they’re going to capture data and how they’re going to evaluate. And we needed some commonality so that if we have three sites, we can compare across the three sites... aggregate all our data together.”
Testing toolkit	Simple tools and methods to enable PHE teams delivering a DHI to test all aspects of the service or product throughout the development journey. The toolkit could include a guide for face-to-face research, approaches to and models for planning and prototyping, functionality for randomizing users, and digital solutions for validating propositions in the market and/or against existing services.	As a digital professional When deciding what to design and how to design it I need to validate service propositions by testing assumptions So that I can be confident in committing resources to develop them	“It’s fundamental to my role...I’m the one who manages [metric company name] and...also the research we conduct with users to define and validate services prior to committing resources developing them. But also to maximize them later, so to use informal or design-led evaluation means to validate, to research, to prove assumptions prior to designing things.”
Development history	A tool for PHE to record the full development history of their DHI project. This will support decision-making, facilitate process evaluation, and enable successful assurance in line with the Government Digital Service pipeline approach. This record would include user needs, decisions and rationale, testing methods and results, information about the underlying technology, and stakeholder mapping.	As an academic evaluator When doing evaluation I need an in-depth understanding of the intervention and the pathway of action So that I can properly evaluate it	“You need a really good understanding of how and why these things are working and what you're changing...which is often much more complex than people gather.”
Data hub	PHE’s data hub provides access to high-quality, accessible, and understandable public health data for providers, academia, and the wider market. Similar to Transport for London’s Open Data, PHE will encourage developers to use PHE’s data feed to solve public health problems in innovative ways. By setting the standard for the metrics in the data hub and promoting collaboration, this data hub may, in the future, allow for longitudinal analysis of DHIs.	As an evaluator When evaluating a health product or service I need access to clean, accessible and linked data from across the health system So that I can do my evaluation	“A lot of their datasets are in silos, they're maybe not using data the most effectively.”
Publish health outcomes	A place for stakeholders to publish and share how DHIs have met or not met desired health outcomes. This promotes collaboration among teams working in similar areas and enables sharing of best practices. Collaboration among PHE, public health professionals, academia, and suppliers working in digital public health aligns with the Government’s Industrial Strategy and the NHS^e^ Innovation Strategy.	As a digital professional When working with PHE I need to understand clearly what clinical data is required So that I can be clear what success or impact looks like and can provide clinical impact	“We moved away from a lot of input and process measures, and balanced it with output and outcome measures, so we know now what impact they're having with individuals that they're working on, particularly their mental well-being, the escalation of demand into adult social care, and how they're embedding into a neighbourhood context more than they used to before.”

^a^JTBD: Jobs To Be Done.

^b^Data from initial semistructured interviews with representatives of target user groups (academic, digital, and public health).

^c^PHE: Public Health England.

^d^DHI: digital health intervention.

^e^NHS: National Health Service.

#### Corresponding Results

See Table 4 for the final seven concepts created to evaluate the DHIs. The concepts integrated perspectives and approaches from all three user groups and were combined into a *scenario of use* for PHE stakeholders. Figure 3 illustrates how the concepts fit together into a proposed Evaluation Service for helping teams evaluate their public health DHIs. Three concepts were prioritized to take forward to the next phase of work: evaluation thinking, evaluation canvas, and testing toolkit. The evaluation thinking concept was taken forward in a separate work stream and is, therefore, not discussed in this paper. Textbox 2 illustrates how one of the final concepts, evaluation canvas, was derived and iterated through the methodology outlined in stages 1 to 5. This is an iterative process with multiple feedback loops rather than a linear one. See Tables S1 and S2 in Multimedia Appendix 1 for how the prioritized concepts, testing toolkit, and evaluating thinking were derived.

**Sample Jobs To Be Done life cycle from primary research (stages 1 and 2) to co-design workshops (stages 3 and 4) to refined concepts prioritized by Public Health England for further development (stage 5) in evaluating digital health interventions.**

**Evaluation Stage**

- Stage 2: setup, information, guidance, and tools

**Theme**

- Evaluation methods

**User Quote**

- “You have a set of tools and paradigms that are going to be suitable for different types of problems. When you're investigating different types of problems, for particular types of stakeholders, which particular types of objectives, or even particular types of technology, or particular stages of innovation, you have to be careful about finding the right thing and not trying to squeeze a round peg into a square hole.”

**Jobs To Be Done**

- As an evaluator
- When I design/set up an evaluation
- I need access to a range of evaluation tools and paradigms
- So that I can choose the one fit for the problem

**Raw Concept From the Co-design Workshop 2**

- Parallel playbook:
  a series of experiments to validate digital health intervention propositions and iterate these to validate impact

**Refined Concept for Further Development**

- Evaluation canvas:
  the evaluation canvas is Public Health England’s validated and accepted portfolio of metrics, tools and methods for evaluating digital health interventions. This canvas is the first step to creating a knowledge base on the effectiveness of digital health in meeting health outcomes and will support decisions on policy, practice, and research.

### Assumption Mapping and Experiment Design

#### Methodology

Assumption mapping and experiment design were performed to understand what needed to be true for each of the prioritized concepts to work in practice within PHE (project objective 5). This means, for example, that end users find the concept understandable, usable, desirable, and feasible within their workflow (project objectives 2 and 3).

For each concept, the project team identified assumptions and articulated a hypothesis in the form of “We believe [insert user] will [action].” This activity helped identify the circumstances in which a hypothesis would be considered true or false and therefore whether the assumptions were correct or erroneous. Experiments were conducted to test each hypothesis, including design artifacts for stimulating feedback. These experiments were a combination of thought experiments and practical tasks.

The outputs (prioritized concepts) from stages 1 to 5 and the subsequently articulated assumptions, hypotheses, and potential experiments (stage 6) formed the input for the paper prototypes (stage 7), which made the experiments tangible for the participants and project team.

#### Corresponding Results

The underlying assumptions and associated hypotheses were mapped for two concepts, evaluation canvas and testing toolkit, and for the proposed Evaluation Service overall. Ten assumptions were mapped with ten hypotheses (Table S3, Multimedia Appendix 1).

### Paper Prototypes

#### Methodology

Testing sessions were held with both individual users and teams (Table 1) running a live DHI. These sessions differed from the semistructured interviews as they tested whole or partial solutions for the service using paper prototypes (project objective 5). These sessions were used to understand (1) all of the requisite steps, (2) the depth of each individual step, and (3) how the steps should be ordered and interrelated. They enabled the project team to iteratively test their understanding and clarify the evaluation approach. For example, to build a project team’s high-level understanding of the potential service (project objective 5), paper cards representing the stages of the emerging Evaluation Service journey were created (see Multimedia Appendix 3, item 2A showing the image of paper cards representing the stages of the emerging Evaluation Service journey used in stage 7, paper prototyping). Teams put these cards in the order that made the most sense to them and added any missing steps (see Multimedia Appendix 3, showing the image of paper cards representing the stages of the emerging Evaluation Service placed in order by user research participant in stage 7, paper prototyping).

Interactive design artifacts were also used to learn more about the individual steps of the Evaluation Service journey. This included testing logic models (conceptual models representing how a product is expected to work) and service blueprints [28] (diagrammatic representations of how the front- and backstage of a service function and dependencies) to learn how DHI teams align on the desired health outcomes and how they assess whether their DHI meets user needs.

The project team turned each of the aforementioned steps into interactive workshop activities to participate in DHI teams. First, they printed the logic model template and put it on the wall, with descriptions for each step (see Multimedia Appendix 3 showing the image of interactive workshop testing the logic model with a DHI team in Stage 7, paper prototyping). The DHI team then went through each step together. The project team observed the DHI team’s conversations and interactions, noting whether the activity made sense to them, the questions they asked, and the group discussion.

The project team conducted a similar interactive workshop with the service blueprint (see Multimedia Appendix 3 showing the image of interactive workshop testing the service blueprint with a DHI team in stage 7, paper prototyping). In addition to describing the user’s needs and actions, the service blueprint can include layers reflecting the service touchpoints and features, as well as the organizational delivery point of view, such as the people, processes, policies, and systems [29] required to bring each stage of the user experience to life.

The service experience tested had both analog (eg, in-person workshops) and digital components (eg, a digital platform). The proposed digital platform, which includes evaluation guidance and printable workshop materials, is referred to as the Evaluation Service. The Evaluation Service idea was tested with users using wireframes to represent the digital experience of a landing page and web pages for each step of the Evaluation Service. This enabled the project team to learn what users expect from a digital platform, as well as their feedback on content and design. The project team turned the outputs of stage 7 into a prototype of a full Evaluation Service, which was the input for stage 8 (proof of concept).

#### Corresponding Results

Seven partial service experience paper prototype sessions were conducted with a range of participants (Table 1). The project team collated insights from each session, presented below, which corresponded to the concepts and hypotheses tested (Table S3, Multimedia Appendix 1).

The outputs of the paper card sorting exercise resulted in ordered steps for evaluating a DHI: building an interdisciplinary team, creating a conceptual model (logic model), creating a detailed service blueprint, selecting relevant indicators against health outcomes, selecting relevant evaluation methods (testing toolkit), and learning how to use selected methods and indicators to evaluate a DHI (testing toolkit and evaluation canvas).

By testing different paper versions of conceptual models (logic models and/or theory of change), the project team learned that the logic model was the simplest tool. Participants expressed the benefits of a logic model: looking at the bigger picture, choosing KPIs, prioritizing important outcomes to aid decision-making, capturing and updating project elements such as objectives, explaining how a DHI worked, and cocreating DHI project value with stakeholders. These sessions also revealed that the more a DHI team presents the bigger picture of what they are trying to achieve, the better the resultant understanding of the team and stakeholders.

These testing sessions demonstrated that participants focused more on measuring the uptake of a DHI than the impact on health outcomes. Teams did not know about national resources, such as PHE’s Public Health Profiles (Fingertips), and so did not link their DHI to nationally recognized health measures. The project team observed that participants would benefit from signposting and guidance in the proposed Evaluation Service to link nationally recognized and validated measures.

Participants preferred the printed workshop format of the logic model rather than a web-based version, as it was easier to collaborate. Participants requested more accessible language and the use of arrows to indicate causality. Participants liked referring to previous or example logic models to see whether their version made sense by comparison. It was suggested that example logic models and guidance should be part of the proposed Evaluation Service offering.

The result of testing the evaluation canvas was breaking it down into its constituent parts and merging it into the Evaluation Service. For example, the program elements were absorbed into the context section of a logic model.

From testing paper and digital versions of the service blueprint, the project team learned that participants responded well to the dimension of time it introduced and the ability to reflect on user experience throughout time as a result of a DHI (not) meeting a user’s needs. By mapping a DHI to its place or impact on a user’s journey (via the service blueprint), participants articulated that they could see the gaps in their understanding of user needs and the features of their DHI. Adding the backstage detail (ie, organizational elements: people, processes, policies, and systems) to the service blueprint also gave participants a better understanding of what their DHI did well and where it could be improved.

Overall, the paper prototype testing revealed that the proposed Evaluation Service should contain information about (refer to Table S3, Multimedia Appendix 1 for details on each hypothesis) the following aspects:

- How to determine the aim of a DHI evaluation with health and service outcomes as core elements of building an evaluation strategy (hypotheses G1, G3, T1, C1, C2, and C3).
- Set up an evaluation at the beginning of DHI development (hypotheses G2.1, G2.2, and T2).
- Process and steps for DHI evaluation (hypothesis T2).
- Constant revision and integration of evaluation strategy into DHI development (hypotheses T1, T2, and C1).
- Who is involved in DHI evaluation (hypotheses T3, C1, and C3).
- Different types of evaluation (impact, process, and health economics; hypothesis C1).
- Selection of evaluation methods and tools (hypotheses T1, T3, C1, and C2).
- Required capabilities and resources (hypotheses G2.1, T1, T3, and C3).
- Barriers that may be encountered during an evaluation (hypotheses C1, C2, and T1).
- External policy and stakeholder factors influencing an evaluation (hypotheses G1).
- Creating a common language and understanding among different disciplines involved in DHI evaluation (hypotheses G3, T1, T3, C1, and C3).

### Proof of Concept: Prototype of the Full-Service Experience

#### Methodology

Stages 1 to 7 culminated in the project team conducting a proof-of-concept test in which a digital team followed the proposed Evaluation Service to build an evaluation approach for their DHI (project objective 5). The proof of concept was a series of workshops run with PHE’s Couch-to-5K app in-house digital team (see Multimedia Appendix 3 showing the illustration of stage 8 proof of concept: prototype of the full-service experience conducted with PHE’s Couch-to-5K app in-house team showing the paper prototyping interactive workshops and associated digital materials). For example, the Couch-to-5K team used the logic model workshop template to define the problem they were addressing and clarify the desired health and service outcomes. They used a service blueprint template to describe users’ needs, experiences, and actions concerning service touchpoints and features, organizational and operational capabilities, and actions, as well as the desired health outcomes.

In addition to testing these sequential design artifacts, an evaluation facilitator role was tested in each workshop with the Couch-to-5K team. The Couch-to-5K team was provided with relevant materials and a brief introduction to the workshop aim and then invited to move through the activity as independently as possible. The Couch-to-5K team was observed to see what made sense to them and what they understood correctly. When the Couch-to-5K team needed help, the evaluation facilitator provided guidance; this helped identify when the guidance or materials were unclear and when a DHI team would need and value facilitator support.

#### Corresponding Results

The logic model workshop brought the Couch-to-5K team together around a shared view of the health outcomes they were trying to deliver for Couch-to-5K app users. By mapping user needs to the service blueprint and aligning them with service features, the Couch-to-5K team obtained a shared view of the user needs and their relation to the desired health outcomes. The Couch-to-5K team identified unmet basic needs (eg, incidents) and areas for improvement in the evaluation journey. The Couch-to-5K digital team was alerted to different evaluation methods and tools relevant to their product and its context (including maturity, budget, time constraints, and risk profile). Spending time reflecting on KPIs highlighted to the Couch-to-5K team additional pertinent indicators worth beyond what they already collected (eg, KPIs related to the broader physical activity market). The Evaluation Service experience subsequently informed the design of the Couch-to-5K behavioral database (incorporating newly identified KPIs aligned to desired health outcomes).

## Discussion

### Principal Findings

PHE collaboratively developed an Evaluation Service with digital developers, academics, and public health professionals involved in the commissioning, design and development, and evaluation of DHIs. Following an iterative UCD approach, which was novel for PHE, the project team worked across and synthesized each discipline’s areas of importance in relation to evaluating DHIs. An in-depth collective understanding of how biomedical and digital evaluation activities map to and can be used during the design and development of a DHI resulted. Testing with PHE’s Couch-to-5K app in-house digital team demonstrated the desirability, viability, and feasibility of the Evaluation Service and led to further investment by PHE.

The primary strength of the work was in PHE’s organizational openness to UCD and service design methodologies that were, at the time, not commonly used by the national body. Although this sparked some cultural tensions, doing so led to knowledge sharing between the external supplier and internal PHE project team and stakeholders, facilitated the synthesis of biomedical and digital evaluation approaches, and grew PHE’s understanding of the benefits of user-centered approaches for designing and evaluating DHIs. Through extensive user and stakeholder engagement throughout the project, we demonstrated to PHE senior leaders and other health system organizations that *design is your friend* and there is a place for design disciplines in public health.

As PHE adapted to the UCD project approach, the project team unearthed tensions among participants such as discomfort with the *fast pace* of the 2-week sprints, divergent expectations on what was considered *good enough* evidence for justifying a user need or concept, and hesitance to try new methods and work in the open. Although some of the PHE stakeholders were initially uncomfortable with the pace and *roughness* of concepts and prototypes, they appreciated that the quick, iterative UCD approach enabled more people to interact, provide feedback, and contribute to the development of the Evaluation Service. The Evaluation Service was thereby informed by, and socialized with, a wider range of professionals involved in DHI development and evaluation. PHE’s senior stakeholders also acquired substantial evidence of the user’s need for the Evaluation Service before requesting further investment from the organization.

### Limitations

We identified three key limitations of this study. First, the sample size in qualitative design research is often small compared with traditional academic qualitative research. This typical design research practice is based on the finding [30,31] that speaking with a representative sample of 5 to 10 people at a time is sufficient to uncover common challenges, understand underlying causes, and inform decisions. In design research and digital development, as outlined by the Government Digital Service [32], the limitation of small sample size is usually overcome by conducting iterative research and increasing the number of users testing and feeding back on the service as it progresses through the initial phases (discovery and alpha) to later phases (beta and live). In this way, the team’s understanding continues to grow as the sample size becomes larger and more diverse with time. The sample size of participants was increased in the later stages of this project; however, this is not within the scope of this paper. Second, PHE’s organizational remit and limited financial and human resources resulted in the underutilization of concepts, with only three of the final seven concepts (Table 4) prioritized for further development by PHE. As shown in the *scenario of use* (Figure 3), the seven concepts complemented each other, and, through the prioritization exercise, the potential of the scenario was not fully tested. Third, substantial time and input were drawn down from members of the working group and relevant PHE senior leaders to upskill service design project team members in evaluating DHIs and share tacit knowledge of evaluating DHIs. This was a strength of the project, as the project team was able to bring a fresh perspective to drawing insight from experts and users as they designed the Evaluation Service; however, the personnel cost must be recognized.

### Conclusions

The potential of DHIs to combine the reach of large-scale population initiatives with the efficacy of individual treatments is yet to be fully realized. It will continue to be unrealized if how we evaluate and use evaluations to inform the iterative design and development of DHIs do not use the perspectives of both biomedical research and digital development. DHIs are an interdisciplinary endeavor, bringing together clinical or population health interventions, digital product development, product and service design, and communication and health promotion. Hence, the evaluation of DHIs is best informed by interdisciplinary approaches to evaluation to understand both the efficacy of a DHI and its usability and desirability, with measures of success that reflect the different stakeholders involved in the commissioning, design, and development of a DHI and its end users [11].

We have outlined the work undertaken by PHE, a national body, to ensure that DHIs contribute to the improvement of population health and that taxpayers receive the most value (improvement to public health) from investment in digital health. The project is a worked example of using UCD, particularly service design, methods to iteratively understand, synthesize, and embed needs and evaluation approaches of both biomedical researchers and digital product developers. Public health’s traditional approach is complemented by the UCD approach, which in turn is made safer and more robust through its interaction with public health. The resultant Evaluation Service enables digital developers or nonacademics to apply evaluation thinking and techniques during the design, development, and implementation of their DHI. By doing so, it demystifies evaluation, traditionally the realm of academia, and harnesses people’s motivations to ensure that their DHI is as good as it can be and improves end users’ health and well-being. PHE has subsequently built a digital version of the Evaluation Service (named *Evaluating Digital Health Products*), which is openly available on the web [33].

## Appendix

Multimedia Appendix 1 Additional figures and tables with examples of artefacts used in the development of the service.

Multimedia Appendix 2 Discussion guide for semistructured interviews.

Multimedia Appendix 3 Images taken during project delivery.

## Abbreviations

DHI: digital health intervention
JTBD: Jobs To Be Done
KPI: key performance indicator
NHSE/I: National Health Service England and Improvement
NICE: National Institute of Health and Care Excellence
PHE: Public Health England
UCD: user-centered design
UCL: University College London
