# Supplementary material for: An Evaluation Service for Digital Public Health Interventions: User-Centered Design Approach
Source: J Med Internet Res. 2021 Sep 8;23(9):e28356. doi: 10.2196/28356 (PMC8459216; doi:10.2196/28356)
Supplement: Multimedia Appendix 1 [file jmir_v23i9e28356_app1.docx]

**Multimedia Appendix 1.** Additional figures and tables with examples of artefacts used in the development of the service.


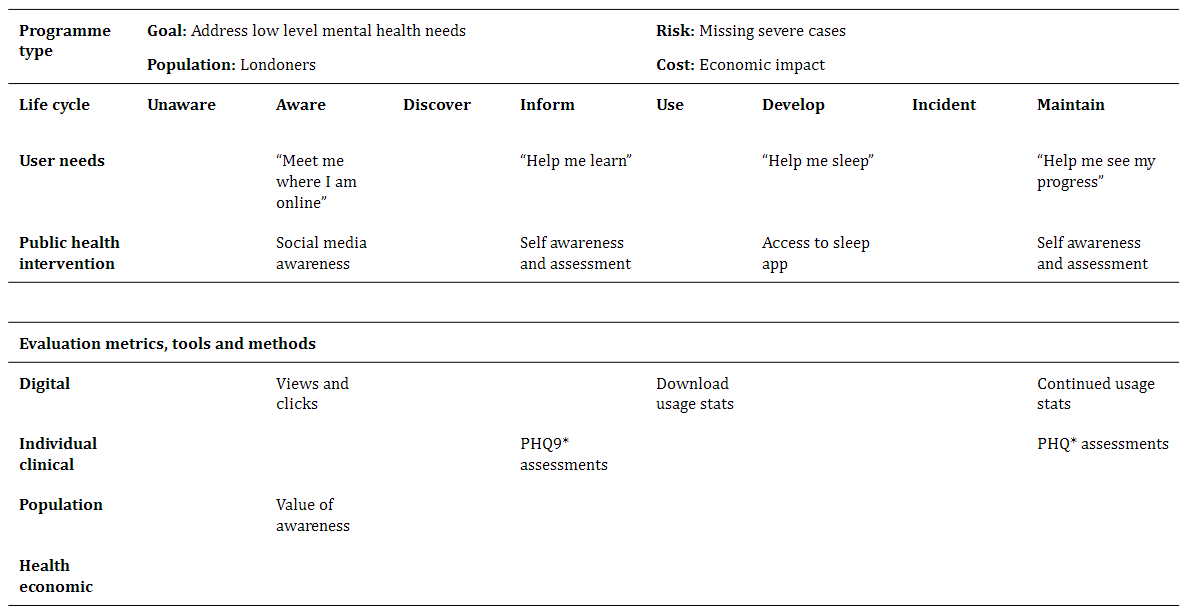


Figure S1. Livework Studio Ltd first visual model combining a real service experience of a digital mental health service, including users’ needs, overlaid with different evaluation methods and metrics that could be integrated into the design and experience of the service. PHQ: Patient Health Questionnaire, where PHQ-9 is a measure for depression.

Textbox S1. Themes identified from semi-structured interviews.

| Access and equality; assessments; authorities and regulatory bodies; change management; clinical risk; collaboration; data analysis and control; data sources; design process; digital health intervention readiness; evaluation methods; evaluations; expectation management; feedback loops; frameworks; funding and costs; intervention type; knowledge management; Key Performance Indicators and metrics; reporting and monitoring; selling and procurement; support and guidance; team and organisational capability; technology; tendering, application and request for proposal; uptake and impact. |
| --- |


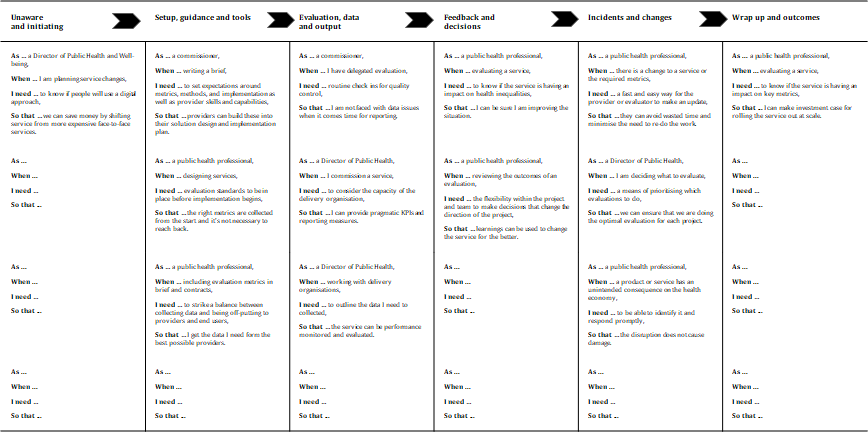


Figure S2. Example Jobs To Be Done for the public health user group going through an evaluation journey that was validated and prioritized at the first co-design workshop. KPIs: key performance indicators.


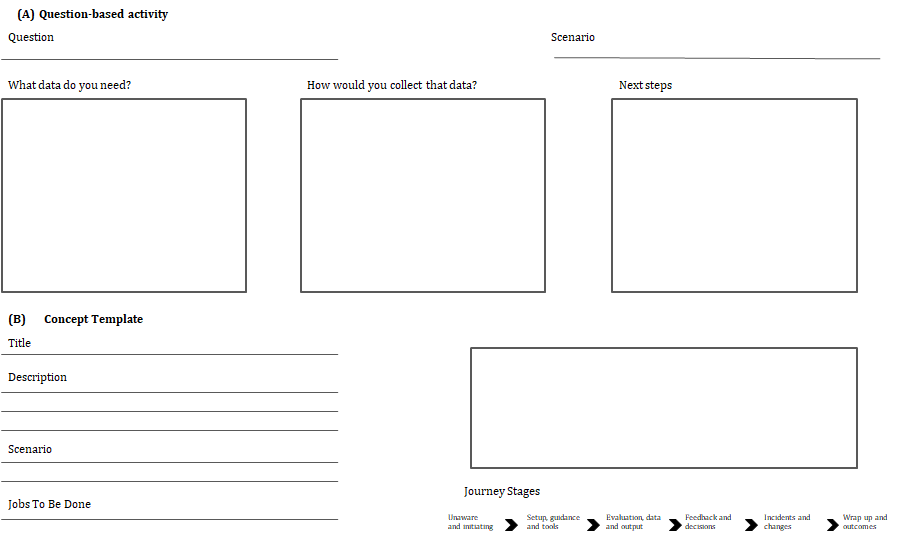


Figure S3. Co-design workshop two design artifacts (A) question-based activity and (B) concept template provided to attendees to stimulate thinking and provide structure for concepts.

Table S1. Sample JTBD life cycle from primary research (stages 1, 2) to co-design workshops (stages 3, 4) to refined concepts prioritised by PHE for further development (stage 5) in evaluating DHIs for prioritised concept Testing Toolkit.

| User type | Digital |
| --- | --- |
| Evaluation stage | Stage 2: set-up, information, guidance and tools; Stage 3: data, decisions and outputs |
| Theme | Evaluation methods |
| User quote | "It's fundamental to my role...I'm the one who manages [metric company name] and…also the research we conduct with users to define and validate services prior to committing resources developing them. But also to maximize them later, so to use informal or design led evaluation means to validate, to research, to prove assumptions prior to designing things." |
| JTBD | As a digital professional  When deciding what to design and how to design it  I need to validate service propositions by testing assumptions  So that I can be confident in committing resources to developing them |
| Raw concepta | Parallel Playbook: a series of experiments to validate DHI propositions and iterate these to validate impact |
| Refined concept for further development | Testing Toolkit: Simple tools and methods to enable PHE teams delivering a DHI to test all aspects of the service or product throughout the development journey.  The toolkit could include a guide for face-to-face research, approaches to and models for planning and prototyping, functionality for randomising users, and digital solutions for validating propositions in the market and/or against existing services. |

a Raw concept from the co-design workshop 2.

Table S2. Sample JTBD life cycle from primary research (stages 1, 2) to co-design workshops (stages 3, 4) to refined concepts prioritised by PHE for further development (stage 5) in evaluating DHIs for prioritised concept Evaluation Thinking (not the subject of this paper).

| User type | Public Health |
| --- | --- |
| Evaluation stage | Stage 1: unawareness and initiating an evaluation |
| Theme | Team and organisational capability |
| User quote | “You have to get them at the beginning. And it's really hard when you don't get them at the beginning, 'cause then you've got to try and do a retrospective evaluation. You never have the right data. It's never as good of quality.” |
| JTBD | As a Director of Public Health  When commissioning services  I need evaluation to be aligned closely with service delivery  So that it is formative and not a separate piece of work |
| Raw concepta | Integrated Evaluation: build in evaluation thinking, skills and tools at the beginning of the DHI development process |
| Refined concept for further development | Evaluation Thinking: When doing a digital health project at PHE, evaluation thinking, skills and tools should be integrated into the project from the start.  Evaluation needs to be a central part of the design process and iterative delivery of any PHE service or product. |

a Raw concept from the co-design workshop 2.

Table S3. Pivotal assumptions and associated hypotheses for the Testing Toolkit concept, Evaluation Canvas concept and proposed Evaluation Service. These concepts were prioritised for further work in stage 5.

| Concept | Assumption | Hypothesis |
| --- | --- | --- |
| Evaluation Service | G1: PHE can show impact on health outcomes for DHIs | We believe PHE will use the Evaluation Service for demonstrating impact on health outcomes for DHIs and use of the Evaluation Service is linked to demonstration of impact |
|  | G2.1: For the Evaluation Service to be effective it needs to be introduced at the beginning of DHI development  G2.2: We will be able to introduce the Evaluation Service into an existing DHI | We believe digital teams can apply the Testing Toolkit at any stage in the development life cycle to ensure their DHI can demonstrate clear and measurable impact in new or in-development or established DHIs |
|  | G3: The Evaluation Canvas and Testing Toolkit need to provide a clear benefit for the user so that they see the value in using it. | We believe users see the benefit in using an Evaluation Service (composed of the Evaluation Canvas and Testing Toolkit) for demonstrating impact on health outcomes. We will know this to be true if digital teams agree to using the Evaluation Service (including through referrals) |
| Testing Toolkit | T1: PHE teams will use the Testing Toolkit | We believe PHE teams will use the Testing Toolkit for applying appropriate and validated tool(s) to improve the quality of a DHI. We will know this to be true if:  1) users use the Testing Toolkit;  2) use of the Testing Toolkit has effect on decisions and development, and  3) more high quality, impactful interventions are developed after use of the Testing Toolkit |
|  | T2: The proposed evaluation approach will fit into the DHI development life cycle | We believe digital teams can apply the Testing Toolkit at any stage in the DHI development life cycle to ensure their DHI can demonstrate clear and measurable impact in new or in-development or established DHIs. |
|  | T3: An “academic expert” isn't needed alongside the Testing Toolkit | We believe digital teams will use the Testing Toolkit successfully without help of an academic to support decision making while designing and implementing an evaluation. We will know this to be true if decisions made by a digital team are validated by academic experts who reach the same conclusion.  We believe use of the Testing Toolkit by digital teams will lead to more efficient use of academic expert time, a more enabled workforce doing more evaluation and academic expertise used to the best advantage. |
| Evaluation Canvas | C1: Users are willing to use the Evaluation Canvas to evaluate the different DHIs | We believe digital teams will use Evaluation Canvas for creating an evaluation strategy. We will know this to be true if:   1. digital teams use the Evaluation Canvas to make an evaluation strategy, and 2. digital teams have the capability to do an evaluation of DHIs. |
|  | C2: The Evaluation Canvas is a useful tool | We believe digital teams will use Evaluation Canvas for creating an accepted evaluation strategy. We will know this to be true if:   1. DHI evaluations by digital teams are rigorous; 2. DHI evaluations by digital teams are validated by the public health and academic community, and 3. digital teams are able to use the outputs of the evaluations to improve product performance. |
|  | C3: The Evaluation Canvas can fit into the workflows of public health professionals, academics and digital developers | We believe digital teams that are multidisciplinary (containing public health and academic professionals) will use Evaluation Canvas throughout the DHI development to support iterative learning, strategy, and overcoming scaling hurdles. We will know this to be true if:   1. digital teams use the Evaluation Canvas 2. use of the Evaluation Canvas has effect on decisions and development, and 3. more high quality, impactful intervention are developed after use of the Evaluation Canvas |
