# Supplementary material for: An Evaluation Service for Digital Public Health Interventions: User-Centered Design Approach
Source: J Med Internet Res. 2021 Sep 8;23(9):e28356. doi: 10.2196/28356 (PMC8459216; doi:10.2196/28356)
Supplement: Multimedia Appendix 2 [file jmir_v23i9e28356_app2.docx]

**Multimedia Appendix 2.** Discussion guide for semistructured interviews.

As the interviews were semi-structured the discussion guide questions (below) are not an exhaustive list of questions asked of interview participants.

Overview

- Talk a little about yourself, your background and your role?
- Do you develop or commission products and services?
- Can you tell us what evaluation means to you?
- Have you ever participated directly in an evaluation of a product or service?
- Would you say evaluation is built into your process for developing products and services?
- Tell us about an evaluation you’ve been involved in.
- How do you normally measure the success of a product or service?
- Can you tell us what your normal evaluation process is? Eg. sprints, randomised controlled trials, alpha/beta testing

| SECTION 1: UNAWARENESS AND INITIATING AN EVALUATION | | | |
| --- | --- | --- | --- |
| Qs | Core questions | Digital | Academic / Public health |
| 1.0 | Thinking about how you got involved in a previous evaluation... | | |
|  | KEYWORDS: UNAWARE, TRIGGERS, DISCOVERY | | |
| 1.1 | How do you find out that an evaluation should take place on a product or service? | Were you aware that an evaluation should happen before you started the project? | How do you find out about evaluations? |
| 1.2 | What factors triggered the need for the evaluation? |  | Were you involved in the design of the evaluation? |
| 1.3 | What stage had the product or service reached when you got involved? |  |  |
| 1.4 | Who were the key stakeholders in the evaluation? |  | Were you doing the evaluation for someone else? |
| 1.5 | Were you involved in the design of the evaluation? | Were there any specific technical considerations? | Were there any specific academic considerations? |
| 2.0 | Thinking about awareness of evaluations in general... | | |
|  | KEYWORDS: LEARN, OBLIGATIONS, FUNDING, CREDIBILITY, EXTERNAL/INTERNAL, COMMUNITIES, GROUPS, PLATFORMS | | |
| 2.1 | How would you expect to become aware that you can or should evaluate a service? | Do you think evaluations integrate well into the digital product development process? | How do you normally get involved in an evaluations? |
| 2.2 | Where would you get information or ideas to measure the success of a product or service? |  | What factors are important to you getting involved in an evaluation? |
| 2.3 | Do you use or belong to any groups, platforms or services that support evaluating products and services? | Are there any useful digital resources that you use or are aware of? |  |
| 2.4 | What is your experience of services you belong to already? What benefits do they provide? | Are there any digital communities, platforms you use to get information or support and advice? | Can you share any theories and or concepts that you subscribe to when taking on evaluations? |
| 2.5 | Do you collaborate or partner with anyone on product and service evaluation? |  | Are there any practicalities around collaboration / partnering that impact on your work? (eg. recruitment) |
| 2.6 | What sort of relationships do you have with collaborators? |  |  |
| 3.0 | When considering an evaluation... | | |
|  | KEYWORDS: KPIs, MEASUREMENT, OBJECTIVES, OUTCOMES | | |
| 3.1 | What measurements / KPIs matter most when considering evaluating a product or service? |  |  |
| 3.2 | What are the practical concerns when considering evaluation as part of your project/role? | Is there anything specific to consider when evaluating digital interventions? | Would you think about evaluating a digital service differently from a non-digital? |
| SECTION 2: SETUP, INFORMATION, GUIDANCE & TOOLS | | | |
| 5.0 | When thinking about setting up an evaluation... | | |
|  | KEYWORDS: SET UP, ROLES, RULES, TOOLS, SUCCESS CRITERIA | | |
| 5.1 | Do you set up evaluations? (If not you, who does?) | What sort of success criteria would you use for a digital service or product? |  |
| 5.2 | How would you plan or scope an evaluation? | Are there any technology considerations when planning an evaluations? | What practical considerations are key when planning evaluations from an academic POV? (funding, research councils) |
| 5.3 | Do you already use guidance for evaluation? | If you do, would you mind sharing it with us? | Where do you find design guidance (Journals, bodies, institutions) |
| 5.4 | Is guidance easy to find and access? | What’s the quality of digital guidance? |  |
| SECTION 4: DATA, DECISIONS & OUTPUTS | | | |
| 6.0 | In terms of collecting, managing, storing data from evaluations... | | |
|  | KEYWORDS: MANAGING, PERMISSIONS, ETHICS, ACCESS, STRUCTURE, STANDARDS, SHARING, SUBMITTING, GDPR | | |
| 6.1 | Can you talk about the types of data that are important in helping you measure success? | Can you talk about your experience of storing, managing and sharing data from a technical perspective? | What are your expectations around data sources and your needs? |
| 6.2 | Can you elaborate on how you access & use this data? | Can you talk about your experience of sharing the data with stakeholders? |  |
| 7.0 | Thinking about analysis and data processing phase... | | |
|  | KEYWORDS: PROCESSING, TOOLS, STANDARDS, METRICS, ANALYSIS | | |
| 7.1 | Can you share a little on your process for analysing and processing your evaluation data? | What apps and tools do you use? |  |
| 7.8 | What's your experience of this approach? | Elaborate on any digital tools you use | Main considerations for analysis (design & completeness of data) |
| SECTION 5: OUTCOMES, FEEDBACK, DECISIONS | | | |
| 8.0 | Thinking about how you make decisions based on outcomes from an evaluation... | | |
|  | KEYWORDS: DECISION MAKING, FUNDING, DEVELOPMENT | | |
| 8.1 | Talk about the kinds of decisions based on information from evaluations of products and services? | Elaborate on your process and how you use data to make decisions. |  |
| 8.2 | What sort of impact or changes do these decisions have on the process? |  | Can you talk about any specific academic decisions made based on the outputs of an evaluation? |
| 9.0 | Can we ask you about feedback on the evaluation process? | | |
|  | KEYWORDS: COMMUNICATION, FEEDBACK, PHASES, PROCESS | | |
| 9.1 | What sort of feedback loops do you expect to exist when evaluating the success of a product or service? |  | Who are the main stakeholders who feedback during an evaluation? |
| 9.2 | Can you describe how you implement and get value from using feedback? | How do you capture feedback on digital products and services? |  |
| 9.3 | Are there any forms of feedback that get in the way of a successful evaluation? |  |  |
| 9.4 | Are there any specific moments in an evaluation where feedback is useful? |  |  |
| SECTION 6: INCIDENTS, CHANGES | | | |
| 10 | Can we talk about how you manage change and incidents during an evaluation?? | | |
| 10.1 | When evaluating, what sorts of problems do you come across? | Do you have an example of change or pivoting and if that was part of an evaluation process? | Are there any specific issues you’ve come across that have impacted on your method (eg. implementation, fidelity) |
| 10.2 | What do you do in those situations? |  | What do you do to mitigate problems? (eg. Hard to reach groups) |
| SECTION 7: WRAP UP, OUTPUTS, OUTCOMES | | | |
| 11 | Thinking about the end stages of an evaluation... | | |
|  | KEYWORDS: OUTPUTS, OUTCOMES, CLOSING, REVIEW | | |
| 11.1 | What outputs would you expect to have at the end of an evaluation? | Elaborate on what happens at the end of an evaluation of a digital product or service? | What are the range of outputs?eg. papers, case studies, reports |
| 11.2 | How do you decide when an evaluation is complete? |  | What’s the ideal outcome from an evaluation? |
| 11.3 | How do you take learnings into a new evaluation? |  | Elaborate on your approach to Standardised measures vs ones you've made up |
| 11.4 | What is your experience of handing over the evaluation process? | Give us some details on handing what documentation you’ve produced at the end of an evaluation? |  |
| 11.5 | Is there a best practice to share / brief new owner of an evaluation? |  |  |

Concluding questions

- Do you think you understand PHE's role in evaluating products and services?
- Are there any questions we should be asking or researching as part of this evaluation project?
