# Supplementary figures and images for: An Evaluation Service for Digital Public Health Interventions: User-Centered Design Approach
Source: J Med Internet Res. 2021 Sep 8;23(9):e28356. doi: 10.2196/28356 (PMC8459216; doi:10.2196/28356)

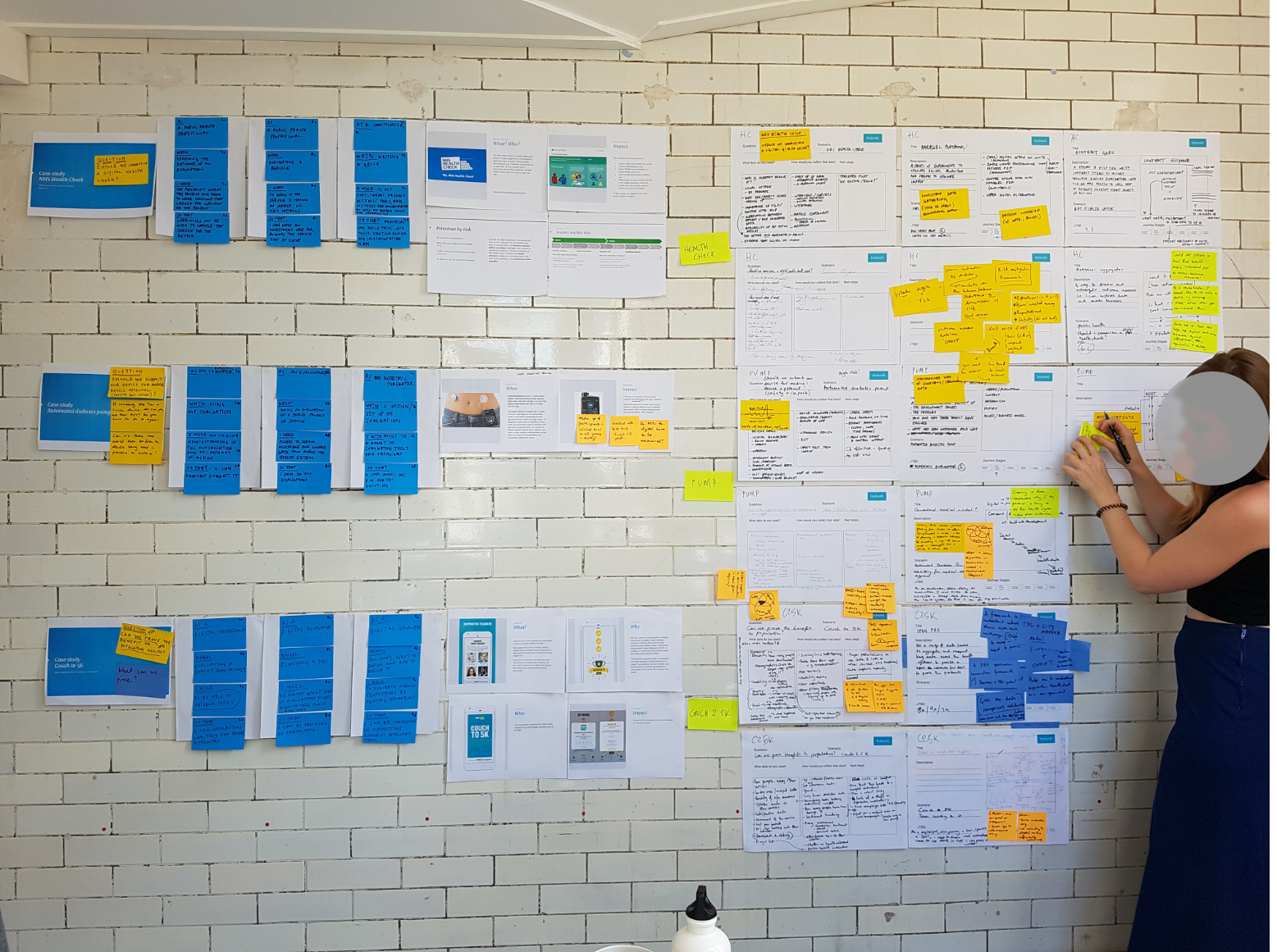

Supplement: Multimedia Appendix 3 [file jmir_v23i9e28356_app3.zip › Item 1_MultimediaAppendix.jpg]

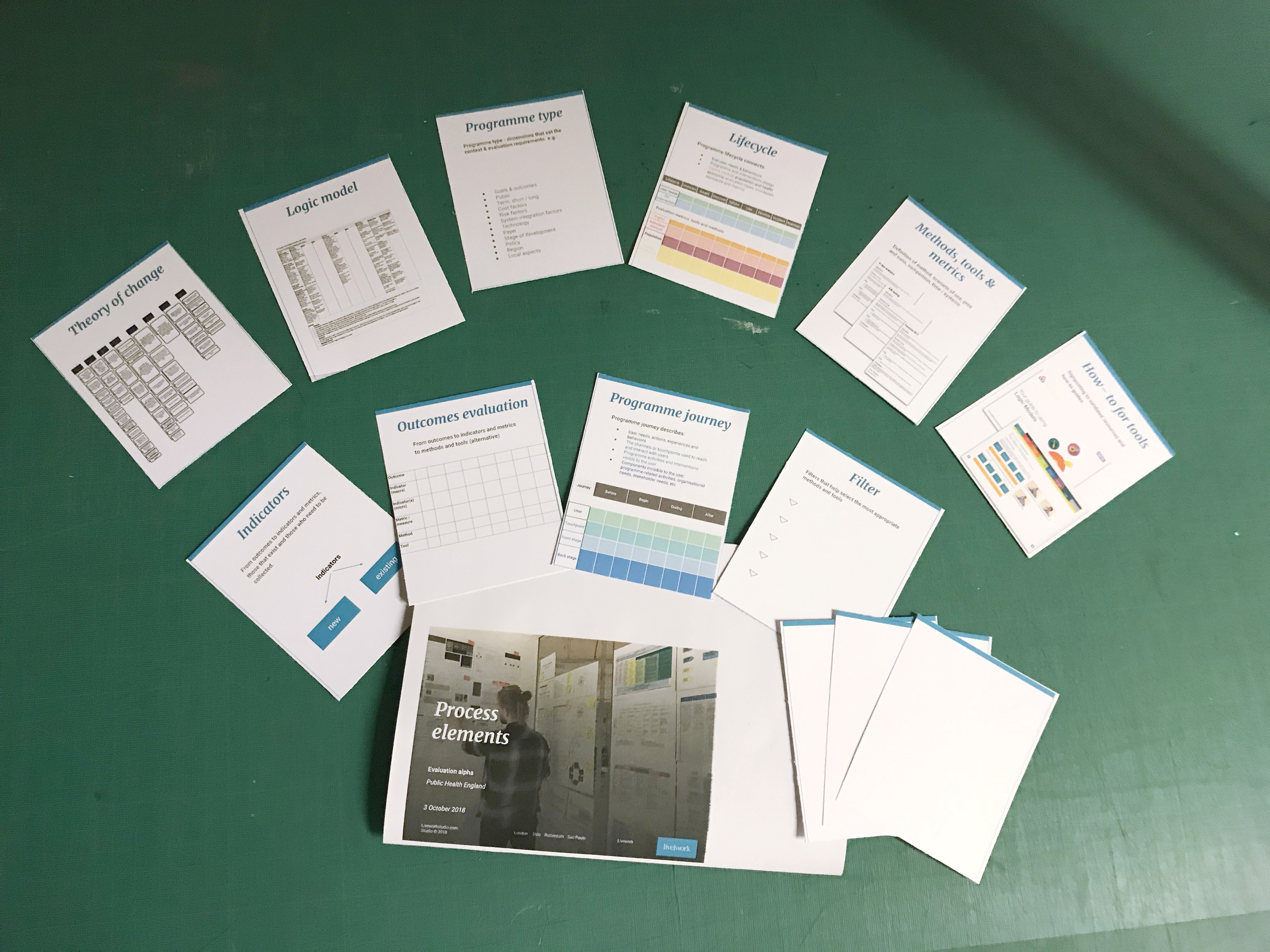

Supplement: Multimedia Appendix 3 [file jmir_v23i9e28356_app3.zip › Item 2A_MultimediaAppendix.JPG]

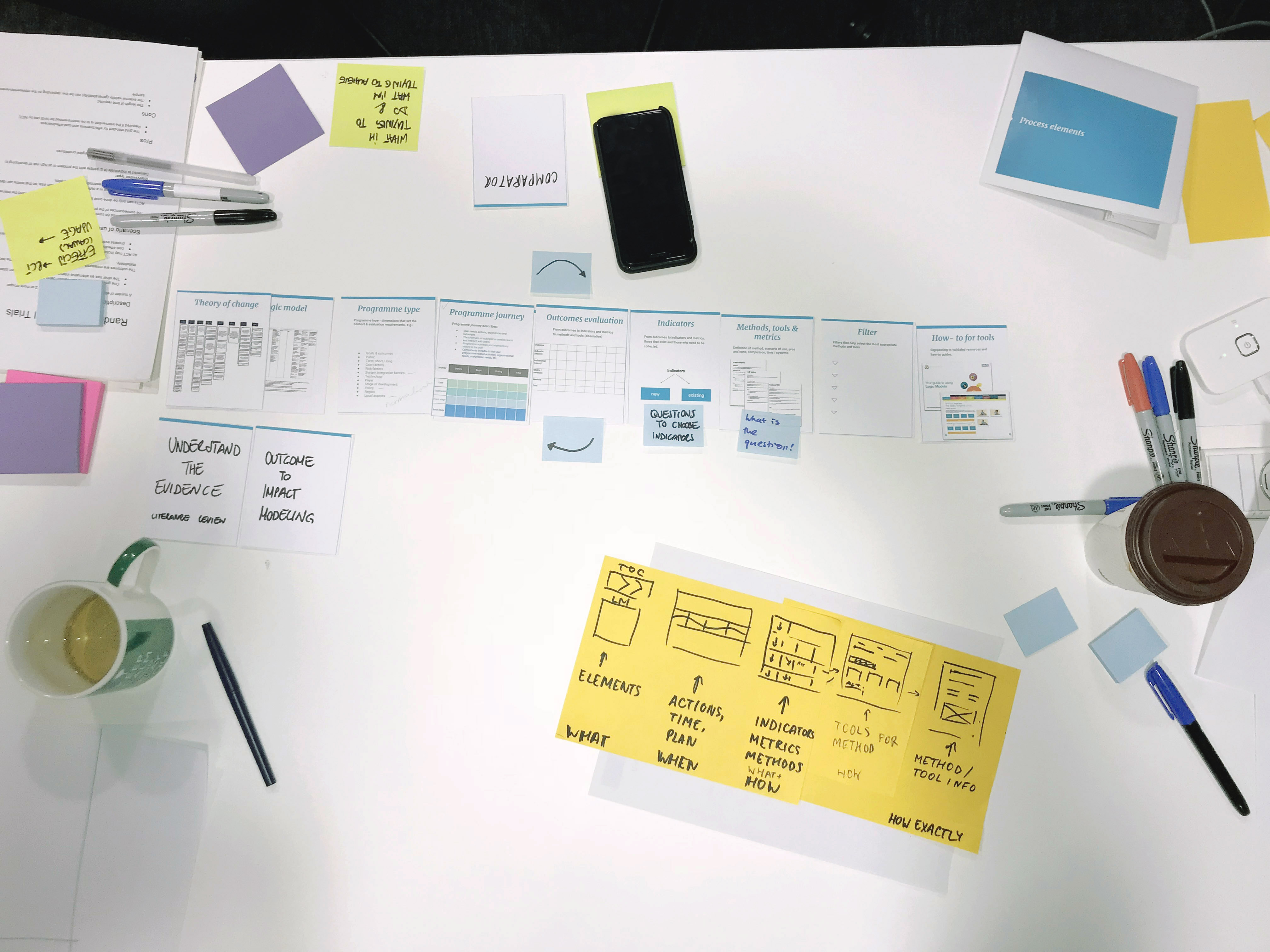

Supplement: Multimedia Appendix 3 [file jmir_v23i9e28356_app3.zip › Item 2B_MultimediaAppendix.JPG]

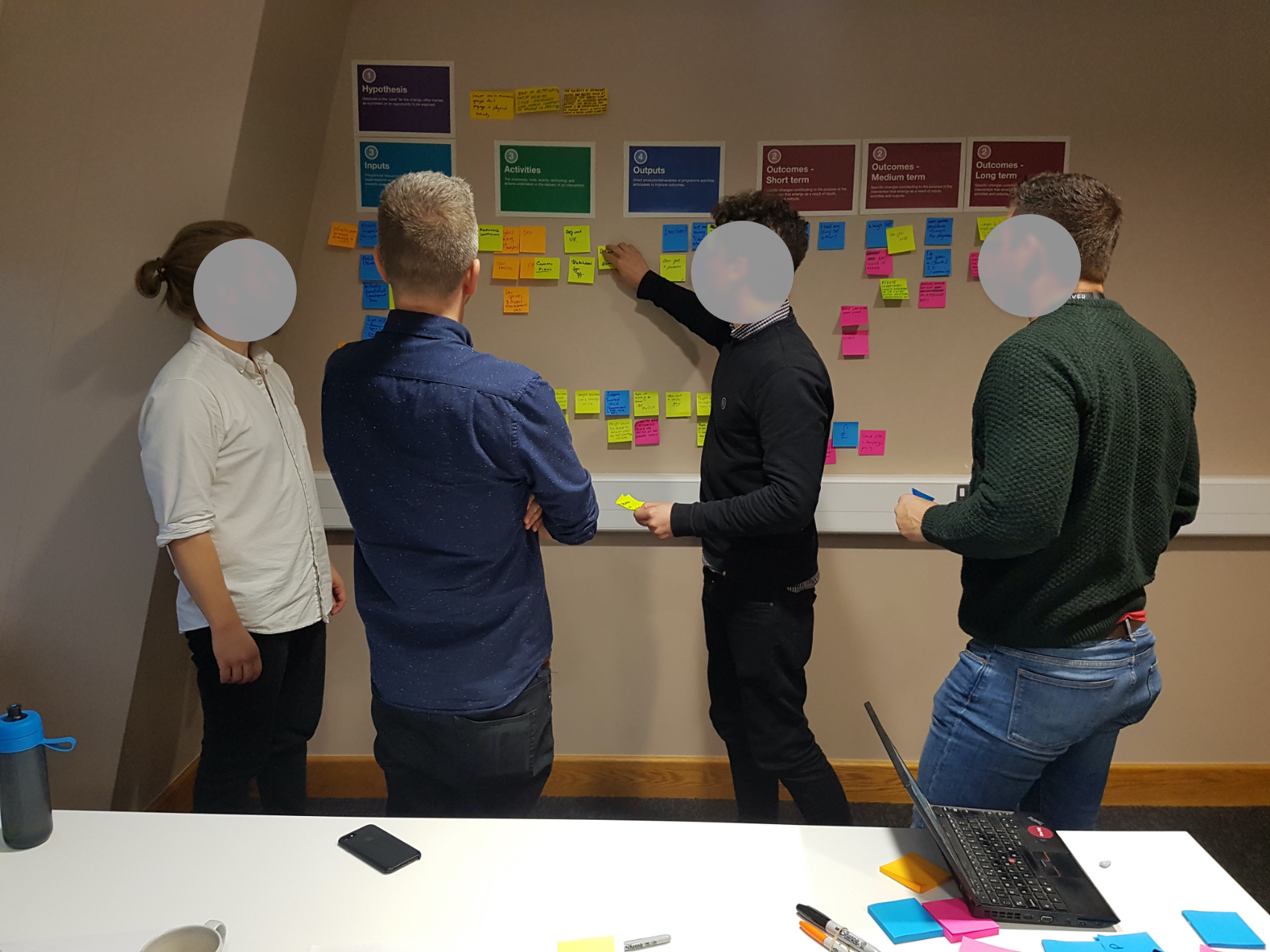

Supplement: Multimedia Appendix 3 [file jmir_v23i9e28356_app3.zip › Item 3_MultimediaAppendix.jpg]

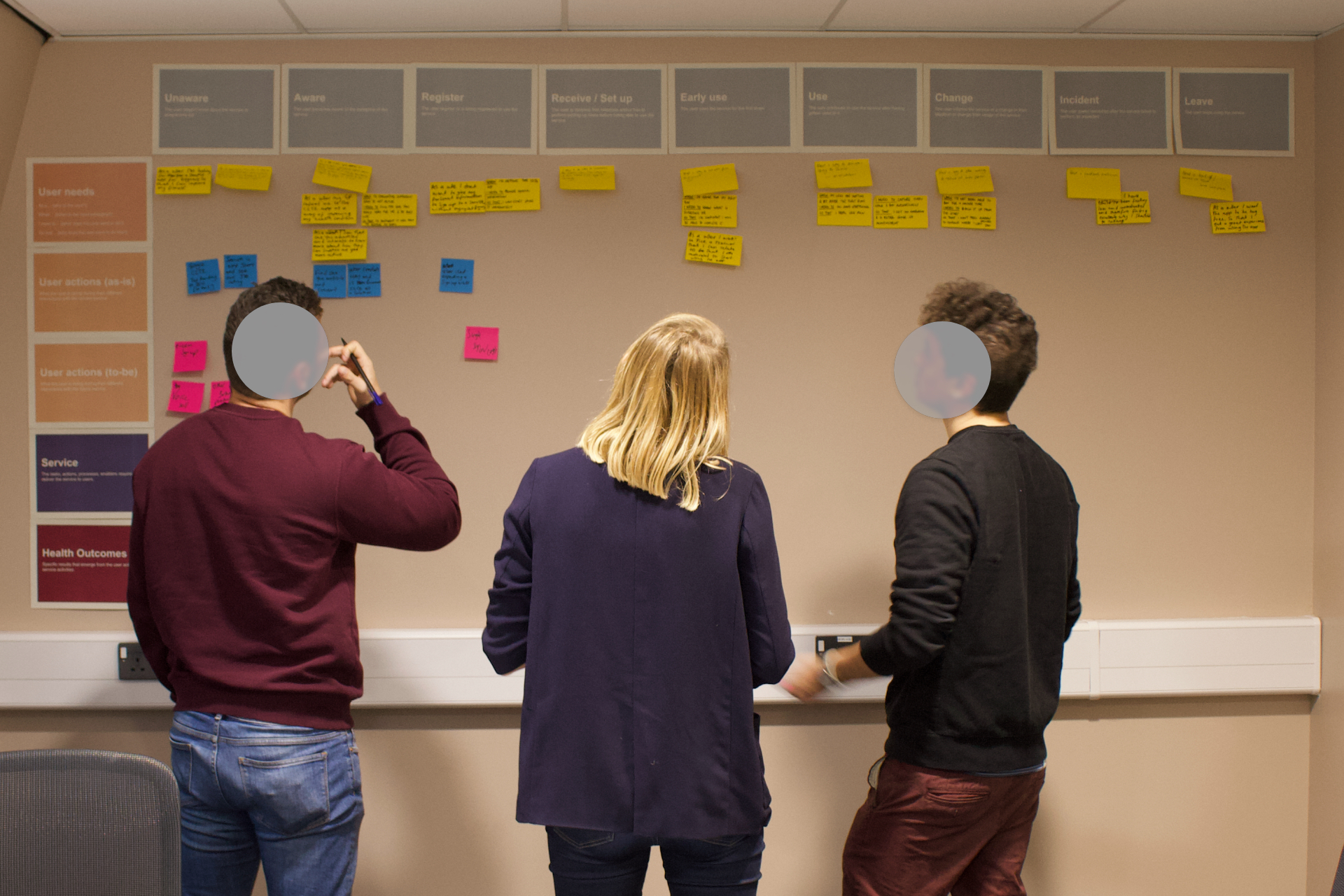

Supplement: Multimedia Appendix 3 [file jmir_v23i9e28356_app3.zip › Item 4_MultimediaAppendix.jpg]

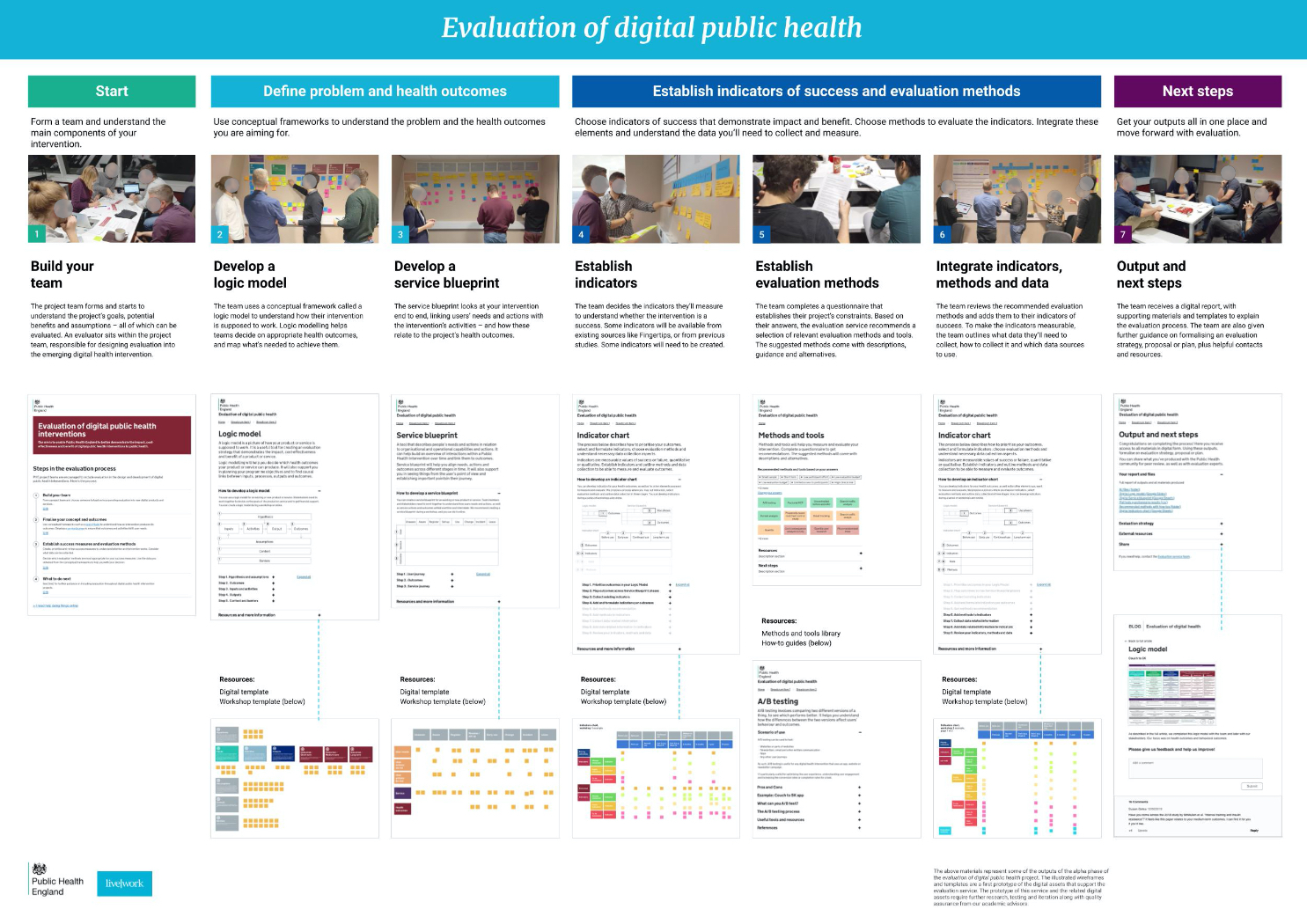

Supplement: Multimedia Appendix 3 [file jmir_v23i9e28356_app3.zip › Item 5_MultimediaAppendix.jpg]
